# Hierarchical Text-Guided Refinement Network for Multimodal Sentiment Analysis

**DOI:** 10.3390/e27080834

**Published:** 2025-08-06

**Authors:** Yue Su, Xuying Zhao

**Affiliations:** School of Mathematical Sciences, Capital Normal University, Beijing 100048, China; y1941611146y@163.com

**Keywords:** multimodal sentiment analysis, semantic alignment, multimodal fusion

## Abstract

Multimodal sentiment analysis (MSA) benefits from integrating diverse modalities (e.g., text, video, and audio). However, challenges remain in effectively aligning non-text features and mitigating redundant information, which may limit potential performance improvements. To address these challenges, we propose a Hierarchical Text-Guided Refinement Network (HTRN), a novel framework that refines and aligns non-text modalities using hierarchical textual representations. We introduce Shuffle-Insert Fusion (SIF) and the Text-Guided Alignment Layer (TAL) to enhance crossmodal interactions and suppress irrelevant signals. In SIF, empty tokens are inserted at fixed intervals in unimodal feature sequences, disrupting local correlations and promoting more generalized representations with improved feature diversity. The TAL guides the refinement of audio and visual representations by leveraging textual semantics and dynamically adjusting their contributions through learnable gating factors, ensuring that non-text modalities remain semantically coherent while retaining essential crossmodal interactions. Experiments demonstrate that the HTRN achieves state-of-the-art performance with accuracies of 86.3% (Acc-2) on CMU-MOSI, 86.7% (Acc-2) on CMU-MOSEI, and 80.3% (Acc-2) on CH-SIMS, outperforming existing methods by 0.8–3.45%. Ablation studies validate the contributions of SIF and the TAL, showing 1.9–2.1% performance gains over baselines. By integrating these components, the HTRN establishes a robust multimodal representation learning framework.

## 1. Introduction

Sentiment analysis (SA) is the task of computationally interpreting human sentiment, including opinions, emotions, and subjective expressions. Traditionally, SA has focused on text, where models infer sentiment based on the distribution and co-occurrence of sentiment-bearing words and phrases [1]. While this approach leverages linguistic patterns to interpret human emotion, it often fails to capture the full richness of real-world affective expressions, which are inherently multimodal.

Speech sentiment analysis incorporates acoustic features such as pitch, energy, and spectral properties. Variations in intonation, rhythm, and voice quality convey subtle emotional signals that are not captured in text alone [2]. Visual sentiment analysis focuses on facial expressions captured in video sequences, where microexpressions, eye blinks, and facial muscle movements serve as non-verbal indicators of sentiment [3]. These modalities exhibit different temporal resolutions: text progresses relatively slowly, audio varies with speech rate, and video captures fine-grained frame-level dynamics. This temporal heterogeneity emphasizes the importance of effective temporal alignment in multimodal systems.

Multimodal sentiment analysis (MSA) refers to systems that integrate and analyze information from multiple modalities (text, audio, and video) to capture complementary signals and improve prediction accuracy. Initially, SA models focused solely on text, but as multimedia content became dominant, the need to understand emotion across modalities became evident. Bimodal and trimodal methods have demonstrated superior performance over unimodal models, as they leverage the complementary nature of non-text signals. Although text is often the most semantically rich modality, it alone is insufficient: emotions conveyed in tone and facial expression can contradict or intensify textual content. For example, sarcasm detection and emotion disambiguation rely heavily on visual and acoustic cues [4,5,6].

The availability of benchmark datasets like CMU-MOSI [7] and CMU-MOSEI [8] has catalyzed the development of MSA models. These datasets consist of aligned video, audio, and text modalities, annotated with fine-grained sentiment scores. However, they also pose significant challenges, including misalignment between modalities, subjective label interpretations, and modality-specific noise. These factors motivate the design of models that can adaptively weigh modality contributions and resolve semantic mismatches between modalities.

Early MSA methods such as the Tensor Fusion Network (TFN) [9] proposed structured multimodal fusion, while models such as MulT [10] introduced crossmodal attention to capture interactions across sequences. Feature-Disentangled Multimodal Emotion Recognition (FDMER) [11] and MISA [12] focused on disentangling modality-invariant and modality-specific features using adversarial learning and crossmodal attention fusion. More recent models like ConFEDE [13] and PS-Mixer [14] improve efficiency and generalization through lightweight fusion and latent interaction modeling. Despite these advances, most existing methods assume modality symmetry or treat all inputs equally, overlooking the fact that one modality, typically text, often dominates sentiment expression.

**Figure 1:**
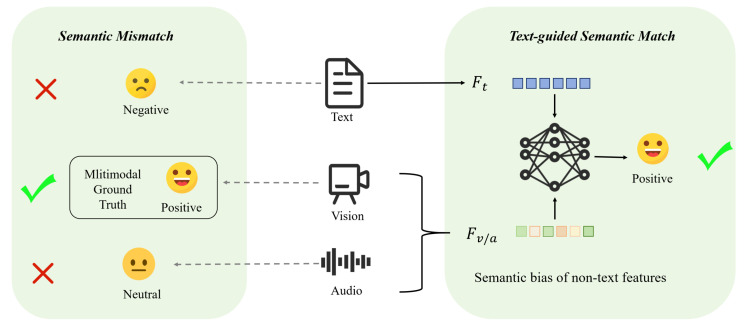
Illustration of multimodal semantic mismatch and our method, which refines text-based predictions using non-text features for better alignment.

In MSA, the text modality typically plays a dominant role, as its sentiment polarity is often more consistent with the overall emotional expression derived from the fusion of all three modalities. In contrast, visual and acoustic modalities can introduce noise and their emotional signals are generally less precise. However, this dominance of text is not universal. Text-Guided alignment systems have the advantage of leveraging rich semantic information from text, but they also face challenges such as text-dominated bias, which can lead to inaccurate sentiment predictions in cases where non-textual cues (e.g., audio and visual signals) carry more relevant emotional information. As illustrated in the left example of Figure 1, the sentiment polarity of the text diverges significantly from the general multimodal sentiment. In such cases, excessive reliance on the text modality during fusion can lead to biased or inaccurate predictions.

By categorizing the three modalities into two groups, the text modality and the non-text modalities (audio and visual), as illustrated in the right example of Figure 1, this approach effectively addresses the identified issue and enhances the accuracy of sentiment prediction. Based on this distinction, we leverage the non-text modalities to guide and refine the sentiment representation of the text modality, thereby enabling the model to generate predictions that better reflect the true emotional expression. This strategy fully exploits the complementary strengths of non-text modalities and enhances the robustness and expressiveness of the HTRN, especially in scenarios involving modality inconsistency.

To address the challenges of modality alignment and redundant information in MSA, the Hierarchical Text-Guided Refinement Network (HTRN) introduces a structured refinement mechanism that leverages multi-scale textual guidance, as illustrated in Figure 1. The HTRN integrates a Shuffle-Insert Fusion module to enhance unimodal feature robustness and a Text-Guided Alignment Layer to adaptively refine non-text modalities through hierarchical semantic features. Furthermore, the gated fusion strategy dynamically regulates modality contributions, ensuring an optimal balance between modality interactions and information retention, ultimately improving sentiment analysis performance. Our experiments show that HTRN outperforms existing multimodal models in terms of accuracy and efficiency, achieving significant reductions in parameters and FLOPs without compromising performance. In summary, the main contributions of this research are as follows:

- We introduce Shuffle-Insert Fusion (SIF) to unimodal features by inserting empty tokens at fixed intervals, disrupting local correlations, and encouraging the HTRN to learn more generalized representations with enhanced feature diversity.
- We utilize a Text-Guided Alignment Layer (TAL) to guide the refinement of audio and visual representations while dynamically adjusting their contributions through learnable gating factors. The TAL ensures that non-text modalities remain semantically coherent, suppressing irrelevant signals while preserving essential crossmodal interactions.

This paper is organized as follows. First, in Section 2, we review recent advances in multimodal sentiment analysis and modality-specific representation learning. Then, in Section 3, we introduce the Hierarchical Text-Guided Refined Network (HTRN), detailing its components: Shuffle-Insert Fusion (SIF), a Text-Guided Alignment Layer (TAL), and a crossmodal fusion mechanism. In Section 4, we evaluate the HTRN on three benchmark datasets, comparing it with state-of-the-art baseline methods and conducting ablation studies to verify its design. Section 4.4 presents quantitative results, efficiency comparisons, and visualizations that demonstrate the interpretability and robustness of the model. Finally, in Section 5, we summarize the contributions of the HTRN, highlight its limitations, and outline future research directions.

## 2. Related Work

### 2.1. Multimodal Sentiment Analysis

Multimodal sentiment analysis has evolved significantly, largely driven by the introduction of benchmark datasets and advances in model design. The Tensor Fusion Network (TFN) proposed in [9] was one of the first models to explicitly model intramodal and intermodal interactions via tensor-based fusion, laying the foundation for structured multimodal representation learning. In their subsequent work, ref. [8], they introduced the CMU-MOSI and CMU-MOSEI datasets, which have since become standard benchmarks for multimodal sentiment analysis. These datasets contain aligned text, audio, and visual data with utterance-level sentiment annotations, enabling fine-grained analysis of multimodal affective expressions under real-world conditions.

In addition to English-language corpora, the CH-SIMS dataset [15] addresses sentiment analysis in the Chinese language, offering fine-grained labels that emphasize consistency and conflicts between modalities. This supports a deeper understanding of how non-verbal cues interact with language in emotionally expressive communication.

Recent research focuses on two primary directions: representation learning and multimodal fusion. For example, models such as MISA [12] decompose each modality into invariant and modality-specific components to better capture discriminative signals, while ConFEDE [13] leverages contrastive learning to separate intramodality and intermodality information. Ref. [16] employs cross-sample fusion to perceive modality-specific information during modal fusion. Similarly, PAMoE-MSA [17] captures polarity-specific and shared features by guiding experts to focus on polarity-aware information, further improving modality representation. AGS-SMoE [18] systematically addresses multimodal competition through causal preemption-based state estimation and adaptive gradient scaling without adding additional parameters, improving the robustness and efficiency of the representation. However, these methods often rely on complex architectures, which can lead to increased computational cost and overfitting due to redundant information.

Fusion-centered methods, including MulT [10] and MAG-BERT [19], design sophisticated mechanisms to align and integrate multimodal inputs. $H^{2}CNA$ [20] introduces heterogeneous hypergraphs to MSA, enabling beyond-pairwise crossmodal interactions through modality-aware graph construction and enhancing robustness via a counterfactual intervention task grounded in causal inference. AtCAF [21] proposes a causality-aware fusion network that models causal relationships using front-door adjustment and counterfactual attention, enabling more accurate multimodal fusion in sentiment analysis. Despite these innovations, the challenge of effectively balancing modality contributions and reducing semantic conflicts remains unresolved. This highlights the need for more robust and efficient models tailored for real-world multimodal sentiment analysis tasks.

### 2.2. Multimodal Representation

#### 2.2.1. Text Modality

With the advancement of deep learning, word embedding techniques have been widely adopted for the extraction of text features. These methods map high-dimensional sparse word representations to low-dimensional dense vectors, capturing contextual relationships and reducing computational complexity [22]. Early models like NNLM [23], HLBL [24], and Word2Vec [25] (including CBOW and Skip-gram) effectively learn lexical patterns, though they depend heavily on large-scale data and under-use global statistics.

Later, GloVe [26] introduced global statistical information in word embeddings, bridging corpus-level co-occurrence and local context. More recently, pre-trained models such as BERT [27] leverage self-attention to capture rich contextual semantics across entire text sequences, enabling more nuanced word representations.

#### 2.2.2. Visual Modality

Visual feature extraction focuses on capturing face-related and motion-based information from videos to analyze emotions. Convolutional neural networks (CNNs) are widely used for this task, eliminating the need for manual feature design. For example, ref. [28] combined local-global features and graph-based inference to enhance facial analysis, while ref. [29] proposed a 3D-CNN approach for efficient extraction of spatiotemporal characteristics.

In practical scenarios, many existing methods rely on publicly available visual feature extraction toolkits, such as OKAO Vision [30], CERT [31], OpenFace [32], and Facet [33]. Specifically, OKAO Vision is designed to identify facial features in each frame, providing indicators such as smile level (on a scale of 0 to 100) and gaze orientation. The Computerized Expression Recognition Toolkit (CERT) automatically extracts various visual cues, including smiling intensity, head movements, facial action units (AUs), emotional states such as happiness and anger, etc. The MultiComp OpenFace 2.0 toolkit offers the extraction of 68 facial landmarks and 17 AUs, as well as detailed data on head pose, orientation, and gaze direction. Similarly, Facet delivers a full pipeline of visual analysis capabilities, including facial keypoint tracking, head posture, eye gaze estimation, AUs, and a histogram of oriented gradient (HOG) features.

#### 2.2.3. Audio Modality

Deep learning is becoming increasingly popular in audio classification, where manually extracted sound features are typically modeled using LSTM and BiLSTM. Inspired by computer vision, ref. [34] used CNNs for automatic extraction of audio features and achieved promising results in sentiment tasks.

In real-world multimodal sentiment analysis applications, researchers often adopt a variety of open-source toolkits, such as OpenEAR [35], openSMILE [36], LibROSA [37], and COVAREP [38]—to extract relevant acoustic features, including MFCC, pitch, energy, and vocal quality. OpenEAR [35] provides an automated extraction pipeline for numerous acoustic parameters, encompassing prosodic, spectral, cepstral, and metrical features, along with the normalization of the speaker through the standardization of the z score. These functionalities are further extended in openSMILE [36], which offers a computation of various low-level descriptors (LLDs), such as Mel-frequency cepstral coefficients (MFCCs), pitch contours, and sound intensity. It also includes higher-level statistical representations such as mean amplitude, RMS energy, and standard deviation. The LibROSA toolkit [37], operating at a sampling rate of 22,050 Hz, facilitates the extraction of frame-level acoustic representations, such as 20 MFCC coefficients and 12 features derived from the Constant-Q Transform (CQT), which are highly indicative of emotional states and vocal intonation. COVAREP [38] improves the set of features with advanced speech signal descriptors, such as MFCCs and glottal source parameters, providing a comprehensive acoustic analysis framework for each spoken expression.

## 3. Method

### 3.1. Overview

**Figure 2:**
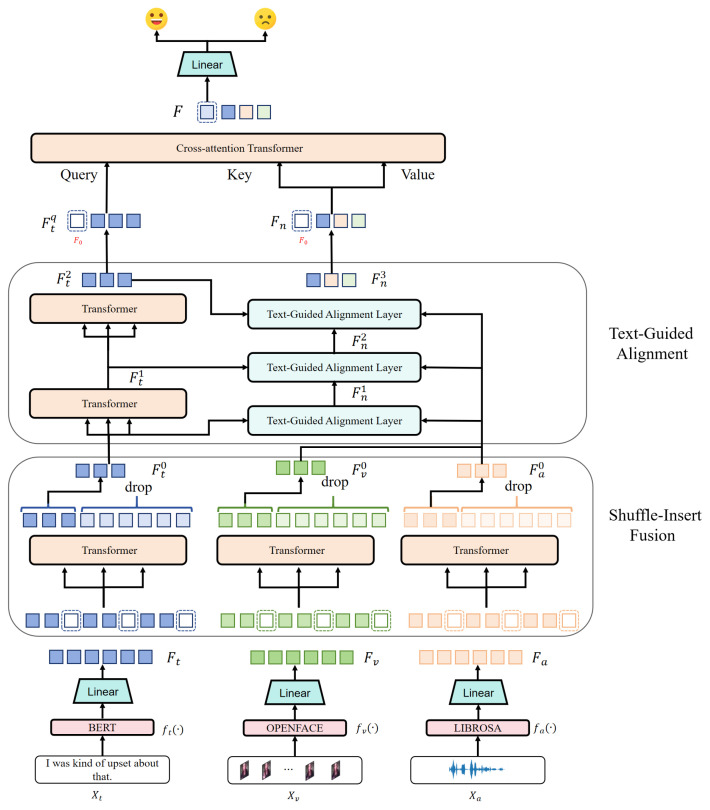
An overview of the HTRN. The HTRN is composed of three core components: (1) Multimodal Embedding, which uses BERT, OpenFace, and Librosa to extract three modal features and then converts them through linear layers and modality-specific transformers. (2) Shuffle-Insert Fusion: The extracted features are processed using independent transformation layers, while tokens are selectively discarded to enhance information diversity. (3) Text-guided alignment: A series of Text-Guided Alignment Layers optimize non-text features based on text features. Finally, the cross-attention transformer aggregates the aligned representations for sentiment prediction.

The model architecture diagram is illustrated in Figure 2. First, a Hierarchical Text-Guided Refined Network (HTRN) extracts modality-specific features from the input data. To mitigate the adverse effects of redundant information, we introduce Shuffle-Insert Fusion (SIF). Then, the Text-Guided Alignment Layer (TAL) uses multi-scale textual features to guide the representation of non-textual features. Finally, the crossmodal fusion transformer fuses the guided non-textual features with the textual features as anchors, thus constructing a hierarchical text-guided optimization network tailored for MSA. Other notations are referenced in Table 1.

### 3.2. Multimodal Embedding

For multimodal inputs, each sample includes text (*t*), audio (*a*), and video (*v*), and the corresponding inputs are indicated as $X_{t}$, $X_{a}$, and $X_{v}$, respectively, where $T_{m}$ (m∈{t,v,a}) denotes the sequence length and $d_{m}$ denotes the vector dimension of each modality. A truncation method is used to unify $T_{m}$ to *T* according to [39,40].

For each modality, precomputed features are extracted using $f_{t}$, $f_{a}$, and $f_{v}$, corresponding to BERT [27], Librosa [37] and OpenFace [32], respectively. These features are standardized to a uniform dimension d=128 using a linear layer:

(1) $$\begin{array}{cc} F_{t} & =\mathrm{Linear}(f_{t}\left(X_{t}\right))\\ F_{a} & =\mathrm{Linear}(f_{a}\left(X_{a}\right))\\ F_{v} & =\mathrm{Linear}(f_{v}\left(X_{v}\right)) \end{array}$$

### 3.3. Shuffle-Insert Fusion

The Shuffle-Insert Fusion (SIF) module aims to enhance feature diversity and robustness by introducing controlled perturbations to generalize the unimodal representation within the unimodal feature space. SIF can reduce redundant information irrelevant to human emotions, achieving higher efficiency with fewer parameters.

Given the representation of characteristics of a specific modality $F_{m}$, where m∈{t,v,a}, we first introduce structured perturbations by inserting null tokens at fixed intervals *I* along the dimension of the sequence length. This operation disrupts the locality of features, prompting the HTRN to learn more general and robust feature representations.

Each modality is then processed independently by the transformer encoder to capture the intramodality contextual information. To balance computational efficiency and information retention, we only extract the first tokens *N*, ensuring that the most valuable features are retained while allowing the subsequent modality fusion mechanism to refine and compensate for missing details adaptively. Finally, these optimized features are integrated into a multimodal fusion framework to construct a more expressive and context-sensitive sentiment analysis model.

### 3.4. Text-Guided Alignment Layer

The Text-Guided Alignment Layer (TAL) aims to leverage rich contextual and abstract meanings embedded in textual information to align non-textual features effectively. The TAL constructs a semantically coherent feature space by utilizing multi-level, multi-scale features extracted from text features, ensuring semantic consistency across modalities. This is particularly crucial for multimodal tasks, where integrating various types of data, such as visual, audio, and textual information, can significantly enhance performance and interpretability.

Attention mechanisms further enhance the TAL, allowing the HTRN to selectively focus on relevant features across modalities and refine crossmodal interactions. Specifically, we adopt scaled dot-product attention [41], defined as follows:

(2) $$\mathrm{Atten}\left(Q,K,V\right)=\mathrm{Softmax}\left(\frac{QK^{T}}{\sqrt{d_{k}}}\right)V$$

where Q,K,V represent *query*, *key*, and *value*, respectively, and $d_{k}$ is the dimension of the *key*. Through attention mechanisms, the HTRN can dynamically prioritize features based on their relevance to the current context, further enhancing the alignment process and facilitating a more subtle understanding of sentiment.

The alignment process first defines features $F_{t}^{0}$ as low-scale language representations, as shown in [40]. The motivation for using hierarchical representations is to capture varying levels of semantic granularity within the text. We generate mid- and high-scale representations by applying two transformer layers to gradually refine the text features, denoted $F_{t}^{1}$ and $F_{t}^{2}$, respectively. This hierarchical structure provides a robust foundation for aligning and matching visual and audio modalities, allowing the model to leverage more complex linguistic features.

**Figure 3:**
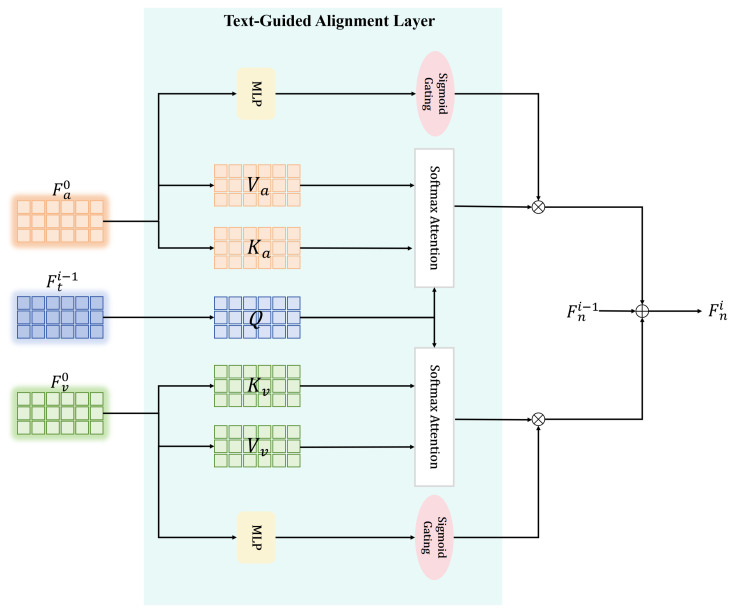
The architecture of the TAL. This module refines the representations of non-text modalities by leveraging semantic cues from textual features. The text feature $F_{t}^{i-1}$ serves as a query to extract corresponding information from both audio ($F_{a}^{0}$) and visual ($F_{v}^{0}$) through softmax attention. The gated factors, computed via sigmoid activation, modulate the contributions of each modality before updating the fused feature representation $F_{n}^{i}$.

As shown in Figure 3, the non-textual features guided by the text features are effectively integrated into the text features through a gated modulation strategy. The gating mechanism adaptively adjusts the contribution of each non-textual mode by computing the gating factors for the audio and video, as follows:

(3) $$g_{a}^{i-1}=\sigma \left(\mathrm{MLP}\left(F_{a}^{0}\right)\right)\in R^{N\times 1}$$

(4) $$g_{v}^{i-1}=\sigma \left(\mathrm{MLP}\left(F_{v}^{0}\right)\right)\in R^{N\times 1}$$

where σ(·) denotes the activation function of the sigmoid. The MLP denotes a single linear layer without activation, followed by a sigmoid function to produce gating weights (the initial $F_{n}^{0}$ is the textual characteristic $F_{t}^{0}$). The motivation for this gating mechanism is to enable the HTRN to focus on the most relevant features from non-text modalities while suppressing noise and irrelevant signals. This selective focus is critical to improve the overall performance of MSA, where certain features may be more significant than others.

Finally, the updated feature representation at the *i*th layer is obtained through a residual fusion mechanism:

(5) $$\begin{array}{cc} F_{n}^{i} & =F_{n}^{i-1}+g_{a}^{i-1}⊙\mathrm{Atten}\left(Q,K_{a},V_{a}\right)\\ \; & \;+g_{v}^{i-1}⊙\mathrm{Atten}\left(Q,K_{v},V_{v}\right) \end{array}$$

Equation (5) ensures that the representation of non-text features effectively integrates non-textual information under the guidance of hierarchical textual features while incorporating modality-specific updates weighted by their respective gates. This mechanism promotes consistency across modalities and allows for adaptive integration of information, leading to better representation and interpretation of sentiments. By integrating text-guided alignment into the multimodal learning process, the model effectively enhances crossmodal consistency, improving the representation and interpretation of sentiments. The TAL implements a structured four-step process to align multimodal features using textual guidance, as formalized in Algorithm 1.
**Algorithm 1:** Text-Guided Alignment Layer (TAL)

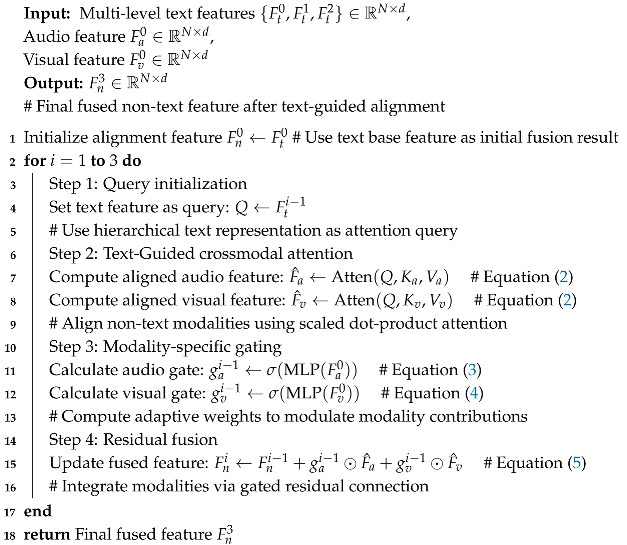


### 3.5. Loss

In the multimodal fusion process, we derive an improved language feature $F_{t}^{2}$ and a refined non-text semantic feature $F_{n}^{3}$. To facilitate information aggregation, we concatenate an initialized token $F_{0}\in R^{1\times d}$ with both $F_{t}^{2}$ and $F_{n}^{3}$, resulting in better representations.

(6) $$F_{t}^{q}=\mathrm{Concat}\left(F_{0},F_{t}^{2}\right)\in R^{(N+1)\times d}$$

(7) $$F_{n}=\mathrm{Concat}\left(F_{0},F_{n}^{3}\right)\in R^{(N+1)\times d}$$

(8) $$F=\mathrm{CrossTrans}\left(F_{t}^{q},F_{n}\right)\in R^{1\times d}$$

Subsequently, we adopt the Crossmodality Fusion Transformer [10,40] to embed both text and non-text information into the learned representations. In this process, the text feature $F_{t}^{2}$ is used as the query, while text-guided transformed non-text feature $F_{n}^{3}$ serves as the key and value, facilitating effective crossmodal interaction. After fusion, the resulting multimodal features are passed through a linear prediction layer to produce the final sentiment estimation ${\tilde{y}}^{\left(i\right)}$. The training objective is formulated as a regression loss over all training samples:

(9) $$L=\frac{1}{B}\sum_{i=1}^{B}{∥{\tilde{y}}^{\left(i\right)}-y^{\left(i\right)}∥}_{2}^{2}$$

where *B* denotes the batch size, $y^{\left(i\right)}$ is the ground truth sentiment score for the *i*-th instance, and ${\tilde{y}}^{\left(i\right)}$ denotes the prediction produced by the HTRN.

## 4. Experiment

### 4.1. Datasets

We evaluated our model on three multimodal datasets: CMU-MOSI [7], CMU-MOSEI [8], and CH-SIMS [15]. (See Table 2).

**CMU-MOSI.** CMU-MOSI contains 2199 opinion-level annotated samples from 93 YouTube vlogs by 89 speakers, with sentiment scores ranging from −3 to 3. The dataset is divided into 1284 training, 229 validation, and 686 test samples.

**CMU-MOSEI.** CMU-MOSEI, the largest dataset for sentence-level sentiment analysis, consists of 23,453 annotated clips from 1000 speakers in 3228 YouTube videos. It is divided into 16,326 training, 1871 validation, and 4659 test samples, with sentiment scores from −3 to 3.

**CH-SIMS.** The CH-SIMS dataset, a Chinese single and multimodal sentiment analysis benchmark, comprises 2281 video clips from 60 raw videos. It contains 1368 training, 456 validation, and 457 test samples, each labeled with sentiment scores ranging from −1 to 1, allowing a complete sentiment analysis.

All experiments were conducted using Python 3.10.4 with the PyTorch 1.2.0 framework accelerated by CUDA 11.4. Model training was performed on a system equipped with an Intel Core i5-12500H processor and an NVIDIA GeForce RTX 3090 GPU (24 GB memory). Due to the varying characteristics of the three datasets, we adopt dataset-specific hyperparameter settings, which are detailed in Table 3.

### 4.2. Evaluation Metrics

We present our results on multiclass classification and regression tasks compared to previous studies [12,15]. For classification, we present results on multi-class accuracy and weighted F1 score. Specifically, we compute accuracy metrics for 2-class (Acc-2), 3-class (Acc-3), and 5-class (Acc-5) predictions on the CH-SIMS dataset, while for CMU-MOSI and CMU-MOSEI, 2-class and 7-class predictions (Acc-7) are evaluated. In addition, two variants of the Acc-2 and F1 score are considered: negative/non-negative (NN, non-exclusion of zero) [9,42] and negative/positive (NP, exclusion of zero) [9,42]. The results of the mean absolute error (MAE) and the Pearson correlation coefficient (Corr) will be provided for regression tasks. Except for MAE, the higher the value, the better the model performs on all metrics.

### 4.3. Baselines

We conducted fair comparisons by reproducing all the leading and state-of-the-art methods on the same experimental platform with identical datasets, evaluation metrics, and environments. The compared methods include the TFN [9], LMF [43], MFM [44], MuLT [10], MISA [12], PMR [45], MAG-BERT [19], FDMER [11], ConFEDE [13], and PS-Mixer [14]. Each of these models represents significant advancements in their respective areas.

**TFN [9]**: The Tensor Fusion Network (TFN) captures crossmodal interactions by constructing unimodal, bimodal, and trimodal features through a 3-way Cartesian product of modality embeddings.

**LMF [43]**: Low-rank Multimodal Fusion (LMF) applies low-rank decomposition to reduce computational overhead while maintaining the effectiveness of multimodal feature fusion.

**MFM [44]**: The multimodal factorization model (MFM) uses unique generative factors for each modality, allowing it to represent modality-specific information and facilitate accurate reconstruction.

**MuLT [10]**: The multimodal transformer (MulT) adopts a bidirectional crossmodal attention mechanism to model interactions between modalities over time, enabling temporal-aware fusion.

**MISA [12]**: Modality-Invariant and Specific Representations (MISA) project modality inputs into two subspaces: a shared space that captures standard crossmodal features and a specific space preserving modality-unique information.

**PMR [45]**: The Progressive Modality Reinforcement Method (PMR) introduces a message hub that iteratively exchanges and reinforces information across unaligned modalities via crossmodal attention to enhance emotion recognition.

**MAG-BERT [19]**: The Multimodal Adaptation Gate Bert (MAG-BERT) is developed by applying a multimodal adaptation gate at different layers of BERT.

**FDMER [11]**: A Feature-Disentangled Multimodal Emotion Recognition method (FDMER) disentangles modality-invariant and modality-specific features using dual encoders with adversarial training and crossmodal attention fusion to achieve refined multimodal emotion representations.

**ConFEDE [13]**: The **Con**trastive **FE**ature **DE**composition Method (ConFEDE) is a unified framework that enhances multimodal sentiment representation by jointly learning contrastive similarity and dissimilarity characteristics in text, video, and audio, with a focus on text-guided alignment.

**PS-Mixer [14]**: The Polar-Vector and Strength-Vector Mixer model (PS-Mixer) is built upon the MLP-Mixer architecture, aiming to improve the interaction between various modal data in MSA.

### 4.4. Results and Analysis

The results of the HTRN on CMU-MOSI and CMU-MOSEI are shown in Table 4, compared to several state-of-the-art baselines. On CMU-MOSI, the HTRN achieves the best performance in all metrics. Specifically, it surpasses the second best method, ConFEDE, by 0.8% on Acc-2 and 0.9 on the F1 score and improves Acc-5 and Acc-7 by 4.9% and 1.6%, respectively. Compared to the TFN and LMF, the HTRN produces lower MAE (0.716) and higher correlation (0.794), reflecting more precise regression and better modeling of multimodal interactions. The TFN and LMF suffer from limited fusion expressiveness or high computational costs, while the HTRN maintains both efficiency and accuracy. Although PS-Mixer achieves competitive classification results, its performance in fine-grained metrics remains less consistent, indicating the HTRN’s advantage in robustness.

On CMU-MOSEI, the HTRN also delivers strong results. It achieves 54.9% on Acc-5, outperforming all competing models. For Acc-2 and F1, the HTRN slightly exceeds PS-Mixer and ConFEDE, showing comparable performance with top-tier methods. Although gains over baselines such as MFM or MISA are smaller than those on CMU-MOSI, the HTRN demonstrates stable improvements and strong generalization. Prior methods focus on static modality fusion or lack effective temporal alignment, while the dynamic token-role design of the HTRN allows for better integration of modality-specific and shared information.

Notably, CMU-MOSI is a smaller dataset with binary sentiment labels primarily based on monologue videos, which makes it prone to overfitting but suitable for observing performance under limited data. In contrast, CMU-MOSEI provides more diverse speakers and topics, along with more sentiment levels (from −3 to +3), thus serving as a more challenging task for model generalization. Our consistent results across both datasets confirm the HTRN’s robustness in both low-resource and large-scale settings.

In addition, to verify the generality of the HTRN, we jointly trained the CMU-MOSI and CMU-MOSEI training sets and evaluated them on their respective test sets. To accommodate the heterogeneity of the two datasets, we designed independent modal embedding linear layers for each dataset while sharing the parameters of the backbone model. Experimental results show that this joint training strategy across datasets effectively improves the overall performance of the two datasets, especially in the classification accuracy and correlation metrics.

Specifically, on CMU-MOSI, the HTRN ^‡^ achieved leading performance in multiple key indicators such as Acc-7, Acc-5, F1, and Corr. Acc-7 improved to 47.2%, and Corr improved to 0.812, indicating that the model has a stronger discriminant ability and a better fitting effect in multi-level classification tasks and affective intensity modeling. It is worth noting that as can be seen from Table 3, the HTRN ^‡^ can achieve faster convergence by simply increasing the batch size to 128 under the same CrossTrans depth and epoch settings as CMU-MOSI, demonstrating good training efficiency and stability. On CMU-MOSI, the HTRN ^‡^ also improved the Acc-5 and Corr metrics, reaching 56.6% and 0.785, respectively, further verifying its strong generalization ability on large-scale multimodal datasets.

This indicates that HTRN can effectively capture the commonalities between CMU-MOSI and CMU-MOSEI despite their topic distribution, sample size, and sentiment expression differences. Driven by the improved multimodal semantic alignment and modeling capabilities, joint training avoids the risk of overfitting to a single dataset. In addition, it improves the model’s generalization ability for sentiment semantics across datasets. This advantage is due to the text-guided dynamic role modeling mechanism, which enables the model to more accurately identify the semantic core in multi-source data, thereby improving downstream sentiment discrimination performance.

We note that on Acc-7 of CMU-MOSEI, neither the single training nor the joint training of the HTRN performed as expected, in contrast to the improvement trend of other metrics. This may be due to the following factors: First, the definition of Acc-7 focuses more on distinguishing the intensity of intermediate emotions, while CMU-MOSEI contains many neutral or mildly emotional expressions, which are more subjective and introduce greater uncertainty in model decisions. Second, since the distribution of emotional labels in CMU-MOSEI is relatively biased towards neutral and light polarities, it is difficult for the model to effectively draw the decision boundary between different categories. To further optimize this indicator, subsequent work may consider introducing a more refined label redistribution mechanism or fuzzy boundary processing for neutral regions to enhance the HTRN’s recognition ability under ambiguous emotional expressions.

As shown in Table 5, the HTRN consistently outperforms all baseline methods on the challenging CH-SIMS dataset across all evaluation metrics. In the fine-grained five-class classification task, the HTRN achieves an Acc-5 of 43.98%, outperforming the closest competitor, LMF, by a significant margin of 3.45 percentage points. Similarly, the HTRN achieves 68. 71% in Acc-3, demonstrating superior capability over early fusion models like the TFN (65.12%) and modality interaction-based approaches like MuLT (64.77%).

Regarding Acc-2, the HTRN demonstrates strong performance by attaining 80.31% accuracy and an F1 score of 80.23, outperforming MuLT (78.56%, 79.66 F1) and MISA (76.54%, 76.59 F1). The HTRN achieves the lowest MAE (0.394) and the highest correlation (0.628), demonstrating improved prediction precision and more substantial alignment with actual sentiment trends.

These improvements can be attributed to the HTRN’s novel hierarchical text-guided alignment strategy, which dynamically guides the crossmodal fusion process and effectively captures fine-grained intermodality dependencies. Furthermore, the SIF module introduces structured perturbations into the fusion process, enhancing the HTRN’s robustness by mitigating modality-specific noise and irrelevant variance. This dual strategy enables the HTRN to preserve semantic consistency while selectively amplifying emotionally salient cues, making it particularly effective across diverse classification levels.

As shown in Table 6, we compare the size and computational complexity of the HTRN with several representative MSA models on CMU-MOSI, excluding the shared BERT-based text encoder for a fair comparison [40,46,47]. Despite its relatively compact architecture with only 2.51 M parameters and 4.76 G FLOPs, the HTRN achieves the highest Acc-2 score of 86.3%, outperforming all baseline models in both efficiency and effectiveness. Compared to ConFEDE, which achieves 85.5% but at the cost of 20.12 M parameters and a heavy computational burden of 134.16 G FLOPs, the HTRN offers an 8× reduction in parameters and 28× reduction in FLOPs, while still improving performance by a notable margin. Even when compared with highly efficient models such as MAG-BERT (1.22 M, 3.91 G) and MISA (3.10 M, 1.7 G), the HTRN achieves a better balance between model size and performance, outperforming them by 1.9% and 2.9% on NP Acc-2, respectively.

These results highlight the efficiency of the hierarchical alignment and structured perturbation strategy of the HTRN, which enables expressive crossmodal representation without incurring significant computational cost. Rather than relying on deep and computationally expensive fusion networks, the HTRN uses a lightweight yet highly effective architecture that selectively integrates multimodal cues, achieving competitive accuracy with significantly fewer resources. This makes the HTRN a promising choice for real-world deployment scenarios where both performance and efficiency are critical.

**Figure 4:**
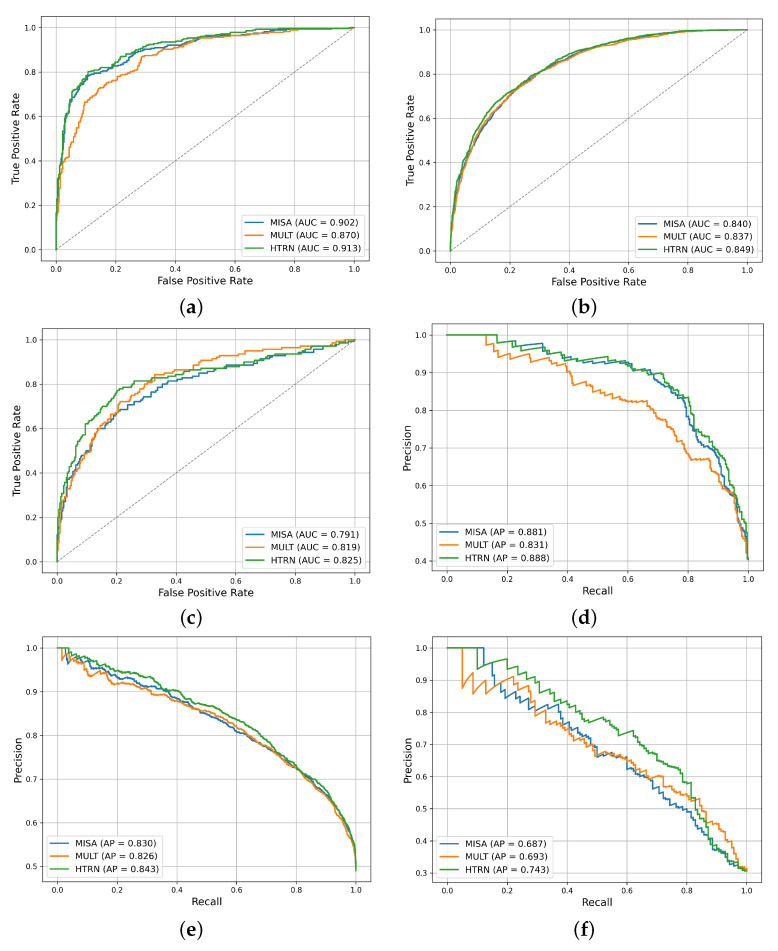
A comparison of ROC and PR curves of different models on three datasets. (**a**) the ROC curve on CMU-MOSI; (**b**) the ROC curve on CMU-MOSEI; (**c**) the ROC curve on CH-SIMS; (**d**) the PR curve on CMU-MOSI; (**e**) the PR curve on CMU-MOSEI; (**f**) the PR curve on CH-SIMS.

Figure 4 compares the ROC and PR curves of MISA, MuLT, and the HTRN on three datasets under the Acc-2 metric. On CMU-MOSI, the AUC value under the ROC curve of the HTRN model reaches 0.913, significantly higher than that of MuLT (0.871) and MISA (0.902), demonstrating better discriminative ability. In the corresponding PR curve, the HTRN also achieves the highest AP value (0.888), outperforming MISA (0.881) and MuLT (0.831).

On CMU-MOSEI, the HTRN demonstrates superior performance, achieving an AUC of 0.849, which slightly exceeds that of MuLT (0.837) and MISA (0.840). The AP value in its PR curve is 0.843, which is also better than that of MISA (0.830) and MuLT (0.826), indicating that the HTRN still has robust sentiment recognition capabilities in complex and diverse natural language contexts.

On the Chinese dataset CH-SIMS, the HTRN achieves an AUC of 0.823, substantially outperforming MISA (0.791) and slightly surpassing MuLT (0.819), demonstrating its robust cross-lingual generalization capability. In the PR curve, the AP value of the HTRN is 0.743, which also leads to MISA (0.687) and MuLT (0.693), further verifying the model’s broad adaptability and strong generalization ability in Chinese MSA.

In general, the HTRN outperforms existing mainstream methods on different datasets and evaluation metrics, especially in the ROC curve and the PR curve, which demonstrated superior classification performance and accuracy, verifying its effectiveness and robustness in MSA.

### 4.5. Ablation Studies

#### 4.5.1. Comparison of Effects of Different Modalities

To evaluate the impact of each modality on the performance of the model and the contribution of the auxiliary modalities to the extraction of text, as shown in Table 7, we conducted ablation studies on CMU-MOSI and CMU-MOSEI, focusing on the metrics NP Acc-2 and Acc-7 on CMU-MOSI, as well as the metrics Acc-5 and MAE on CMU-MOSEI.

For clarity in notation, we designate the text-guided approach in the TAL (Text-Guided Alignment Layer) as TG, with corresponding abbreviations for audio-guided and video-guided methods: AG (audio-guided) and VG (video-guided), respectively. Table 7 indicates that the HTRN further demonstrates that text-guided feature alignment plays the most significant role compared to non-text modalities. As shown in Table 7, TG consistently outperforms AG and VG in both the CMU-MOSI and CMU-MOSEI datasets. Specifically, TG achieves the highest Acc-2 (86.3%) and Acc-5 (54.9%) scores, as well as the lowest MAE (0.531), indicating its superior ability to capture features relevant to sentiment.

In addition, the results highlight the greater contribution value of the audio modality, suggesting that the audio provides more auxiliary information than the visual modality. Specifically, removing the audio input results in a 2.1% drop in NP Acc-2, whereas removing the video input leads to a minor decrease of 0.9%, highlighting the crucial role of the audio modality in the HTRN.

#### 4.5.2. Textual Feature Scale Analysis

In Table 8, we analyze how the textual features on different hierarchical scales affect the performance of the HTRN on CMU-MOSI. The experimental results clearly illustrate the effectiveness of the progressive guidance strategy adopted in the HTRN. Specifically, we observe that each level of textual representation ($F_{t}^{0}$, $F_{t}^{1}$, and $F_{t}^{2}$) contributes differently to the performance, revealing their unique roles in guiding the alignment of non-text modalities. To further validate this observation, we enable different scales of textual features to guide the fusion of non-textual features in the experiments. For layers that do not require language guidance, we replace the TAL with a simple MLP layer. As the experimental results show, enabling hierarchical textual guidance significantly improves performance, confirming the importance of multi-scale semantic cues in the fusion process.

The low-level text representation $F_{t}^{0}$, which captures shallow and local semantics, already provides a strong baseline performance (accuracy 84.5%, F1 score 84.8%). However, its ability to handle complex or abstract expressions is limited. Performance remains competitive when using the mid-level representation $F_{t}^{1}$, indicating its capacity to generalize contextual patterns. Similarly, the high-level representation $F_{t}^{2}$ excels in capturing abstract and global semantics, as evidenced by the strong correlation score (0.782), suggesting its advantage in guiding the global alignment of audio and visual signals.

More importantly, as we progressively combine multiple scales of textual features, the model consistently improves across almost all metrics. For example, combining $F_{t}^{0}$ and $F_{t}^{2}$ leads to a better correlation (0.781), while the combination of $F_{t}^{0}$ and $F_{t}^{1}$ yields the lowest MAE (0.715), demonstrating that complementary semantic cues from different layers are beneficial. The complete integration of the three levels, $F_{t}^{0}$, $F_{t}^{1}$, and $F_{t}^{2}$, achieves the best overall performance with 86. 3% precision, 86.4% F1 score, and a correlation of 0.794. This confirms the importance of multi-scale textual guidance in building a semantically coherent multimodal feature space.

In summary, these results empirically substantiate the fundamental principle underlying our text-guided alignment design. Hierarchical textual features offer increasingly abstract and complementary cues that progressively refine the alignment of non-textual modalities. The HTRN can dynamically bridge the semantic gap between modalities by leveraging such multi-scale representations, leading to more accurate and robust sentiment predictions.

#### 4.5.3. Impact of Shuffle Token Insertion Positions

Table 9 compares the different insertion positions of the shuffle token (interval insertion, front insertion, end insertion, and no insertion setting) on CMU-MOSI and CMU-MOSEI. As shown in Table 9, inserting shuffle tokens at fixed intervals (HTRN) outperforms other strategies across both datasets, achieving the highest Acc-2 (86.3% for CMU-MOSI, 86.4% for CMU-MOSEI) and lowest MAE. This improvement stems from balanced local perturbation: uniformly distributed empty tokens preserve sequential coherence while enhancing feature diversity, similar to structured dropout. Additionally, truncating to the first N tokens compresses redundant information, forcing the model to focus on discriminative features of the early stages.

In contrast, front-/end insertion disrupts sequence integrity by overloading perturbations in localized regions (e.g., erasing initial cues in front insertion), leading to context fragmentation and higher MAE. The baseline without insertion suffers from overfitting to noisy segments in full-length sequences (50 tokens), highlighting the necessity of structured regularization. Thus, the HTRN’s interval-based design optimally balances robustness and efficiency.

#### 4.5.4. Visualization of Token Importance in SIF

**Figure 5:**
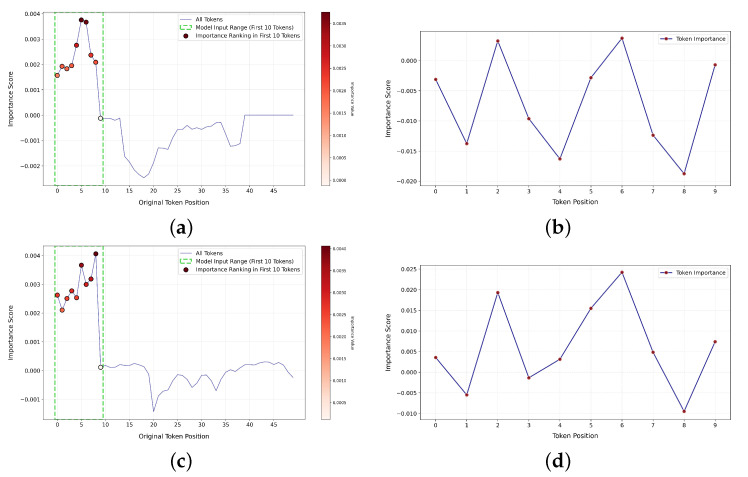
Comparison of visual token importance for different samples on CMU-MOSI. (**a**) Overall visual token importance for sample 1; (**b**) importance of $F_{v}^{0}$ for sample 1 (top 10 tokens); (**c**) overall visual token importance for sample 2; (**d**) importance of $F_{v}^{0}$ for sample 2 (top 10 tokens).

Figure 5 presents our analysis of the visual modality input of the HTRN on CMU-MOSI. We adopt the Integrated Gradients (IG) method to measure the importance of each visual token to the final prediction and visualize the results to verify the model’s attention regions and decision basis when processing video modality information.

In Figure 5, we analyze the first two samples from the CMU-MOSI test set, focusing on all visual tokens ($F_{v}$) before applying the SIF module, as well as the top 10 tokens ($F_{v}^{0}$) retained by the HTRN after using the SIF module. Figure 5a,c illustrate the important distribution of all visual symbols for each sample. The most important positions are highlighted with red dots, and the relative importance is indicated by the intensity of the color. The x-axis represents the position of the tokens in the original sequence, while the y-axis shows the corresponding importance scores. We compute the overall importance of each token by adding the attributions in its feature dimensions using the IG method. In particular, within the interval of the first 10 tokens—marked by green dashed boxes—the importance scores are significantly higher than those of other positions. This suggests that the HTRN tends to focus more on the early part of the input sequence.

To further explore the attention distribution of the HTRN within the selected input range, subplots (b) and Figure 5b,d show the importance scores of each marker in $F_{v}^{0}$. The results show that the HTRN’s attention to these 10 tokens is significantly selective, rather than evenly distributed. This phenomenon supports the design concept of using the SIF module in the HTRN to retain key information and suppress redundant information. It should be noted that the HTRN does not assign the same weight to all frames. Instead, through the SIF module, the HTRN automatically learns the top 10 most representative markers $F_{v}^{0}$, which more effectively represent the core information in the mode and significantly reduce redundant interference, further verifying the effectiveness of the SIF mechanism in information compression and key content extraction.

Moreover, comparing the two samples reveals a degree of dynamic attention distribution. The high-importance token positions within the top 10 tokens are not identical across samples; for example, the model focuses on tokens 0, 2, and 6 in sample 1. In contrast, it shifts attention to tokens 1, 3, and 6 in sample 2. This variation reflects the model’s adaptability to different inputs, showing its ability to flexibly adjust attention based on contextual information and make more semantically aligned multimodal inferences.

In general, Figure 5 not only uncovers the token utilization pattern of the model in the visual modality but also provides an effective tool for interpreting multimodal fusion strategies. The IG-based analysis successfully identifies the key inputs driving the model’s decisions. It supports our hypothesis that effective crossmodal fusion can be achieved using only the top 10 visual tokens. This insight offers strong evidence for the lightweight and efficient design of the HTRN.

#### 4.5.5. Visualization of Attention in TAL

**Figure 6:**
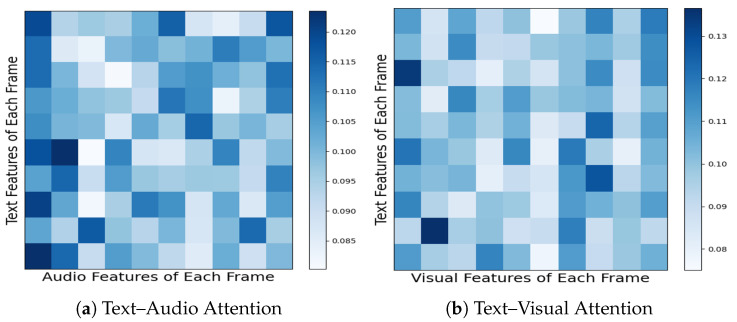
Average attention weights from the final TAL layer.

In Figure 6, we show the average attention heatmaps for the last layer of the TAL on CMU-MOSI. These heatmaps visually interpret how the HTRN attends to different modalities during the matching process. It can be seen that the attention weight assigned to the audio modality is consistently higher than that of the visual modality. This indicates that the HTRN tends to rely more on audio features when constructing a fused multimodal representation, which can be attributed to the audio containing richer and more direct affective cues, such as variations in rhythm, tone, or pitch, providing valuable information for MSA.

To further validate this observation, we refer to the results shown in Table 7, where a modality deletion experiment is conducted. Specifically, we compare the performance degradation of the HTRN when the audio or visual modality is removed. The results show that excluding audio leads to a more significant performance degradation in multiple metrics (classification accuracy, F1 score, and correlation) compared to removing the visual modality. This quantitative evidence is consistent with attention analysis and confirms the central role of the audio modality in the HTRN.

The attention visualization results and the removed modality’s results further confirm this conclusion: Compared with the visual modality, the audio modality in the HTRN provides more auxiliary and emotion-related information. By adaptively focusing on the modality with more information, the HTRN effectively uses the most relevant signals for alignment and prediction, a key factor in its superior performance in MSA.

#### 4.5.6. Visualization of Different Representations

**Figure 7:**
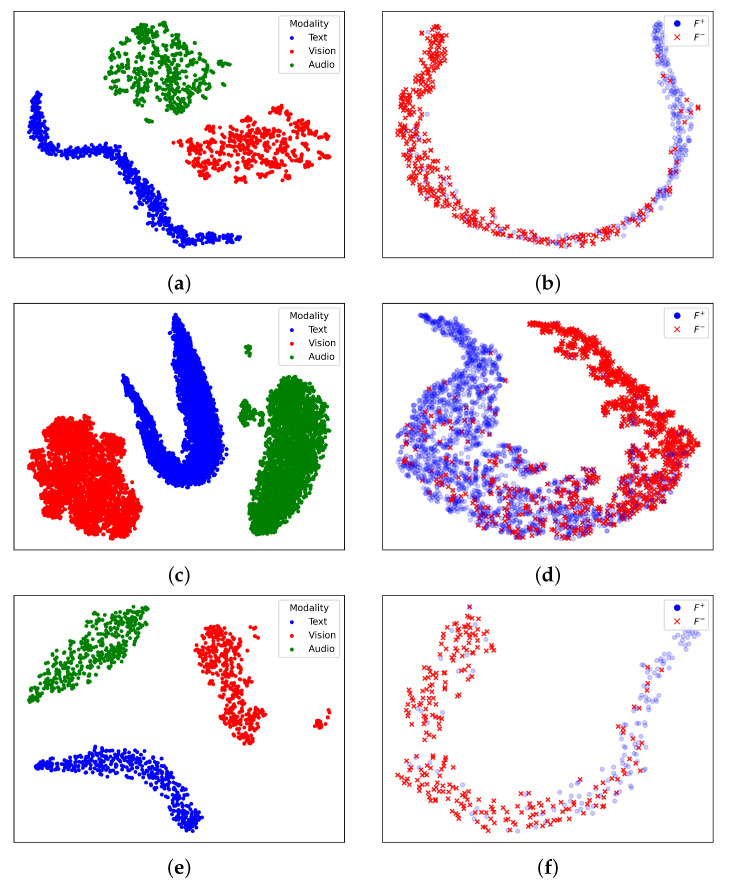
A visualization of different feature representations using t-SNE on three datasets. (**a**,**c**,**e**) presents the unimodal features $F_{t}^{0}$, $F_{v}^{0}$, and $F_{a}^{0}$, where text, vision, and audio are shown in blue, red, and green. (**b**,**d**,**f**) depicts the learned multimodal representations F, with positive ($F^{+}$) and negative ($F^{-}$) samples in blue and red. The visualization demonstrates the HTRN’s ability to learn highly separable representations across different input types and datasets. (**a**) Visualization of trimodal features on CMU-MOSI; (**b**) visualization of F on CMU-MOSI; (**c**) visualization of trimodal features on CMU-MOSEI; (**d**) visualization of F on CMU-MOSEI; (**e**) visualization of trimodal features on CH-SIMS; (**f**) visualization of F on CH-SIMS.

Figure 7 illustrates the visualization of different feature representations using t-SNE on three datasets. The left column of Figure 7 presents the initial unimodal characteristics $F_{t}^{0}$, $F_{v}^{0}$, and $F_{a}^{0}$ extracted from the text, vision, and audio modalities, where each modality forms a distinct group, indicating their separability in the original feature space. The right column of Figure 7 shows the distribution of the learned multimodal representations *F*, where the positive samples ($F^{+}$) are marked in blue and the negative samples ($F^{-}$) in red. The clear separation between the two groups suggests that the model effectively captures discriminative information, facilitating better classification. This visualization highlights the importance of integrating multimodal information to enhance the representation learning process.

#### 4.5.7. Convergence Performance

**Figure 8:**
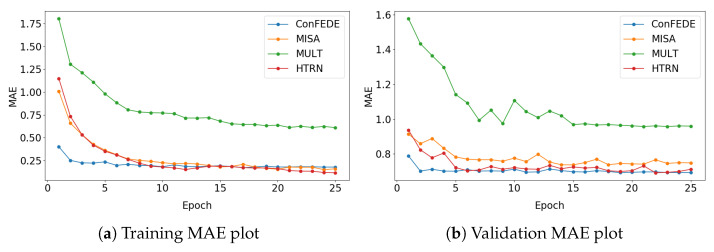
Comparison of convergence on CMU-MOSI.

To further explore the convergence behavior of the HTRN, we present a comparative analysis of MAE training and validation curves on CMU-MOSI alongside several representative baselines, including MuLT, Self-MM, MISA, and ConFEDE. As shown in Figure 8, the HTRN demonstrates the fastest convergence speed on the training set and achieves consistently lower validation errors with reduced oscillations, reflecting its strong generalizability. The HTRN demonstrates the fastest convergence speed on the training dataset among all methods. On the validation dataset, it consistently achieves lower metrics and exhibits more excellent stability. These characteristics demonstrate its exceptional ability to achieve efficient and reliable convergence.

Convergence, in the context of multimodal fusion networks, refers to the stability and reliability of model optimization under various combinations of modes. Poor convergence often indicates overfitting or unstable gradient flow, especially when complex attention-based fusion mechanisms are involved. In contrast, the HTRN benefits from its hierarchical text-guided alignment and structured modality perturbation, which regularize the fusion process and guide the optimization toward more stable and semantically meaningful representations.

The convergence curves visually illustrate that the HTRN reaches an optimal state more efficiently and robustly than its counterparts, highlighting that the proposed design improves performance and improves training reliability, a crucial factor for real-world deployment.

#### 4.5.8. Real Case

**Figure 9:**
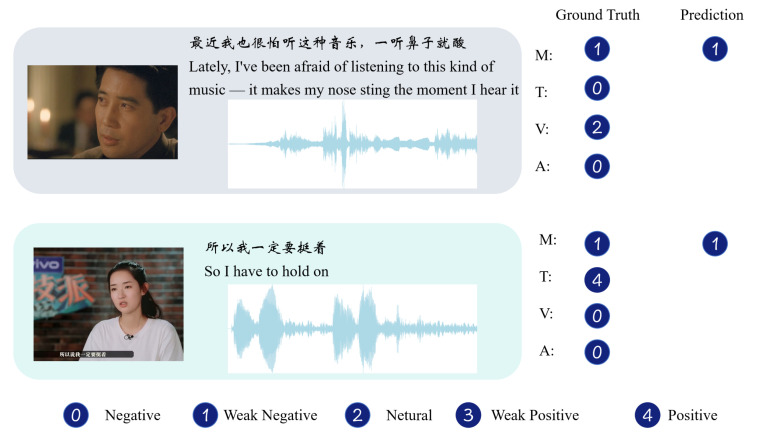
Two examples from the test set of CH-SIMS. “M” denotes the multimodal sentiment label of the sample, whereas “T”, “V”, and “A” correspond to the sentiment labels from the individual modalities: text, visual, and audio, respectively.

To further validate the effectiveness of the HTRN, we present two representative samples from the CH-SIMS test set in Figure 9. In the HTRN, the text modality (T) serves as the primary source for sentiment prediction, while audio (A) and visual (V) modalities act as auxiliary sources. The TAL module allows the auxiliary modalities to modulate the textual sentiment representation, especially when non-verbal cues convey emotions that are not explicitly expressed in text. Additionally, our SIF module effectively minimizes redundant information to optimize the retention of informative features within each individual modality.

In the first sample, the speaker reflects on an emotional reaction to music. The overall ground truth sentiment is Weak Negative (M: 1), mainly driven by the visual modality (V: 2), while both text and audio are labeled as neutral (T: 0, A: 0). Despite the lack of clear sentiment in the transcript, the HTRN correctly predicts a Weak Negative sentiment (M: 1), demonstrating its ability to leverage non-verbal cues such as facial expressions to compensate for the neutrality in text.

In the second sample, the speaker expresses determination and optimism. The text modality shows a strongly positive sentiment (T: 4), but both audio and visual cues are neutral (A: 0, V: 0). The overall sentiment label, however, is Weak Negative (M: 1), which our model accurately predicts. This indicates that the model is capable of detecting inconsistencies between the excessively positive textual content and the comparatively muted emotional cues present in the audio and visual modalities, thereby mitigating textual bias and achieving alignment with the speaker’s true emotional state.

## 5. Conclusions

The HTRN framework achieves significant performance advantages through the synergistic design of the Text-Guided Alignment Layer (TAL) and Shuffle-Insert Fusion (SIF). Compared to audio-guided (AG) and video-guided (VG) methods, text-guided fusion (TG) plays a dominant role in crossmodal alignment, significantly outperforming other strategies. This confirms the critical role of textual semantics in multimodal fusion. The SIF module further enhances feature diversity by inserting blank tokens, effectively compressing redundant information, and highlighting essential features. Furthermore, the HTRN strikes a breakthrough balance between parameter efficiency and computational effectiveness. Compared to larger models such as ConFEDE, the HTRN achieves an 8× reduction in parameter size and a 28× reduction in FLOPs, significantly reducing both parameter size and computational cost. This demonstrates the advantages of its lightweight design while maintaining high performance.

Despite these improvements, two limitations remain: (1) text-dominated bias leads to poorer performance in irony detection, indicating an insufficient use of non-textual cues; (2) fixed insertion intervals have limited adaptability to variable-length inputs. Future work will focus on improving the performance of the HTRN in low-resource scenarios and enhancing its adaptability and generalization capabilities across varying data scales. In addition, we plan to explore more flexible insertion mechanisms and introduce learnable strategies for key information retention, enabling more effective removal of redundant information while preserving critical content.

## Figures and Tables

**Table 1:** Notations.

Notation	Description	Notation	Description
Input and Feature Extraction			
$X_{t}$	Raw input for text modality	$X_{a}$	Raw input for audio modality
$X_{v}$	Raw input for visual modality	$T_{m}$	Sequence length of modality *m*
$d_{m}$	Original feature dimension of modality *m*	*T*	Unified sequence length
$f_{t}\left(\cdot \right)$	BERT-based text feature extractor	$f_{a}\left(\cdot \right)$	LibROSA-based audio feature extractor
$f_{v}\left(\cdot \right)$	OpenFace-based visual feature extractor	*d*	Projected feature dimension (d=128)
$F_{t}$	Projected text features $\in R^{T\times d}$	$F_{a}$	Projected audio features $\in R^{T\times d}$
$F_{v}$	Projected visual features $\in R^{T\times d}$	*I*	Token insertion interval in SIF
Fusion and Alignment			
$F_{t}^{0}$	Text features after SIF	$F_{a}^{0}$	Audio features after SIF
$F_{v}^{0}$	Visual features after SIF	*N*	Number of tokens retained after SIF
$F_{t}^{0:2}$	Hierarchical text features (low/mid/high)	MLP	Multi-Layer Perceptron
*Q*	Query in attention mechanism	*K*	Key in attention mechanism
*V*	Value in attention mechanism	$d_{k}$	Dimension of key in attention
$g_{a}^{i}$	Gating factor for audio modality	$g_{v}^{i}$	Gating factor for visual modality
$F_{n}^{i}$	Refined non-text feature at layer *i*	σ(·)	Sigmoid activation function
Atten(·)	Scaled dot-product attention		
Output			
$F_{0}$	Initialized [CLS] token $\in R^{1\times d}$	$F_{t}^{q}$	Text features concatenated with $F_{0}$
$F_{n}$	Non-text features concatenated with $F_{0}$	*F*	Final fused feature $\in R^{1\times d}$
${\tilde{y}}^{\left(i\right)}$	Predicted sentiment score	$y^{\left(i\right)}$	Ground truth sentiment score
L	Regression loss (MSE)	*B*	Batch size

**Table 2:** The detailed statistics of CMU-MOSI, CMU-MOSEI, and CH-SIMS.

Datasets	CMU-MOSI	CMU-MOSEI	CH-SIMS
Train	1284	16,326	1368
Valid	229	1871	456
Test	686	4659	457
Language	English	English	Chinese
Range of labels	[−3,3]	[−3,3]	[−1,1]

**Table 3:** Hyperparameters for datasets.

Hyper-Parameters	CMU-MOSI	CMU-MOSEI	CH-SIMS	CMU-MOSI and CMU-MOSEI
I	5	5	5	5
N	10	10	10	10
TAL Depth	3	3	3	3
CrossTrans Depth	2	3	4	2
Batch Size	64	128	64	128
Initial Learning Rate	6 $\times \;10^{-5}$	6 $\times \;10^{-5}$	2 $\times \;10^{-5}$	2 $\times \;10^{-5}$
Optimizer	AdamW	AdamW	AdamW	AdamW
Epochs	50	100	100	50
Warm Up	✓	✓	✓	✓
Cosine Annealing	✓	✓	✓	✓

**Table 4:** A comparison of the HTRN with other state-of-the-art approaches on CMU-MOSI and CMU-MOSEI. Boldface highlights the best performance. Results marked with * are reported by [12], while those marked with ^†^ are from [39]; “-” indicates that the corresponding metric was not reported in the original paper; ^‡^ indicates that the HTRN is trained on a dataset that combines the CMU-MOSI and CMU-MOSEI training sets, and the results are tested on their respective test sets. Metrics labeled with ↑ indicate better performance with higher values, and those labeled with ↓ indicate better performance with lower values.

Method	CMU-MOSI						CMU-MOSEI					
	Acc-7 ↑	Acc-5 ↑	Acc-2 ↑	F1 ↑	MAE ↓	Corr ↑	Acc-7 ↑	Acc-5 ↑	Acc-2 ↑	F1 ↑	MAE ↓	Corr ↑
TFN * [9]	34.9	-	-/80.8	-/80.7	0.901	0.698	50.2	-	-/82.5	-/82.1	0.593	0.700
LMF * [43]	33.2	-	-/82.5	-/82.4	0.917	0.695	48.0	-	-/82.0	-/82.1	0.623	0.677
MFM * [44]	35.4	-	-/81.7	-/81.6	0.877	0.706	51.3	-	-/84.4	-/84.3	0.568	0.717
MulT [10]	40.0	-	-/83.0	-/82.8	0.871	0.698	51.8	-	-/82.5	-/82.3	0.580	0.703
MISA [12]	42.3	-	81.8/83.4	81.7/83.6	0.783	0.761	52.2	-	83.6/85.5	83.8/85.3	0.555	0.756
PMR [45]	40.6	-	-/83.6	-/83.4	-	-	52.5	-	-/83.3	-/82.6	-	-
MAG-BERT [19]	43.6	-	82.4/84.4	82.5/84.6	0.727	0.781	52.7	-	82.5/84.8	82.8/84.7	0.543	0.755
FDMER [11]	44.1	-	-/84.6	-/84.7	0.724	0.788	54.1	-	-/86.1	-/85.8	0.536	0.773
ConFEDE [13]	42.3	-	84.2/85.5	84.1/85.5	0.742	0.784	**54.9**	-	81.7/85.8	82.2/85.8	0.522	0.780
PS-Mixer [14]	44.3	-	80.3/82.1	80.3/82.1	0.794	0.748	53.0	-	**83.1**/86.1	**83.1**/86.1	0.537	0.765
MulT ^†^ [10]	-	42.7	-	-	-	-	-	54.2	-	-	-	-
MISA ^†^ [12]	-	47.1	-	-	-	-	-	53.6	-	-	-	-
HTRN	**45.9**	**52.0**	**84.4/86.3**	**84.5/86.4**	**0.716**	**0.794**	52.9	**54.9**	**83.1/86.4**	82.7/**86.5**	0.531	0.778
HTRN ^‡^	**47.2**	**52.9**	**84.6/86.3**	**84.6/86.3**	**0.689**	**0.812**	54.7	**56.6**	83.0/**86.7**	82.6/**86.8**	**0.521**	**0.785**

**Table 5:** A comparison of the HTRN with other state-of-the-art approaches on CH-SIMS. Note: Boldface highlights the best performance. Results marked with ^†^ are from [39]. “-” indicates that the corresponding metric was not reported in the original paper. Metrics labeled with ↑ indicate better performance with higher values, and those labeled with ↓ indicate better performance with lower values.

Method	Acc-5 ↑	Acc-3 ↑	Acc-2 ↑	F1 ↑	MAE ↓	Corr ↑
TFN ^†^ [9]	39.30	65.12	78.38	78.62	0.432	0.591
LMF ^†^ [43]	40.53	64.68	77.77	77.88	0.441	0.576
MFM ^†^ [44]	-	-	75.06	75.58	0.477	0.525
MuLT ^†^ [10]	37.94	64.77	78.56	79.66	0.453	0.564
MISA ^†^ [12]	-	-	76.54	76.59	0.447	0.563
MAG-BERT ^†^ [19]	-	-	74.44	71.75	0.492	0.399
HTRN	**43.98**	**68.71**	**80.31**	**80.23**	**0.394**	**0.628**

**Table 6:** Comparison of model parameters and computational complexity. Note: Boldface highlights the best performance. Metrics labeled with ↑ indicate better performance with higher values, and those labeled with ↓ indicate better performance with lower values.

Method	Parameters ↓	FLOPs ↓	Acc2 on CMU-MOSI ↑
MuLT [10]	2.57 M	32.9 G	83.0
MISA [12]	3.10 M	**1.7 G**	83.4
MAG-BERT [19]	**1.22 M**	3.91 G	84.4
ConFEDE [13]	20.12 M	134.16 G	85.5
HTRN	2.51 M	4.76 G	**86.3**

**Table 7:** Comparison of the effects of different modalities on CMU-MOSI and CMU-MOSEI. Note: Boldface highlights the best performance. Metrics labeled with ↑ indicate better performance with higher values, and those labeled with ↓ indicate better performance with lower values.

Method	CMU-MOSI		CMU-MOSEI	
	Acc-2 ↑	Acc-7 ↑	Acc-5 ↑	MAE ↓
TG	**86.3**	44.3	**54.9**	**0.531**
AG	84.8	45.2	54.3	0.534
VG	84.6	44.2	53.9	0.544
w/o A	84.2	45.9	54.1	0.544
w/o V	85.2	**46.9**	54.6	0.537

**Table 8:** The impact of different scales of textual features on model performance on CMU-MOSI. Note: Boldface highlights the best performance. Metrics labeled with ↑ indicate better performance with higher values, and those labeled with ↓ indicate better performance with lower values.

$F_{t}^{0}$	$F_{t}^{1}$	$F_{t}^{2}$	Acc-2 ↑	F1 ↑	MAE ↓	Corr ↑
✓			83.1/84.5	83.3/84.8	0.730	0.774
	✓		83.7/85.3	83.4/85.2	0.737	0.767
		✓	83.2/85.3	83.1/85.3	0.733	0.782
✓		✓	82.3/83.2	82.2/83.2	0.729	0.781
✓	✓		82.7/85.1	82.6/85.2	**0.715**	0.783
	✓	✓	83.1/84.8	83.5/84.7	0.722	0.777
✓	✓	✓	**84.4/86.3**	**84.5/86.4**	0.716	**0.794**

**Table 9:** Comparison of effects of shuffle token insertion positions on CMU-MOSI and CMU-MOSEI. Note: Boldface highlights the best performance. Metrics labeled with ↑ indicate better performance with higher values, and those labeled with ↓ indicate better performance with lower values.

Method	CMU-MOSI		CMU-MOSEI	
	Acc-2 ↑	MAE ↓	Acc-2 ↑	MAE ↓
HTRN	**86.3**	**0.716**	**86.4**	**0.531**
Front insertion	85.8	0.736	85.8	0.537
End insertion	85.0	0.738	85.0	0.542
No insertion	85.5	0.720	85.2	0.533

## Data Availability

All data supporting this research are from previously reported studies and datasets, which have been cited within the article. The CMU-MOSI dataset is available for download through http://multicomp.cs.cmu.edu/resources/cmu-mosi-dataset/ (accessed on 8 June 2025). The CMU-MOSEI dataset, recognized as one of the largest publicly available MSA datasets, is available for download through http://multicomp.cs.cmu.edu/resources/cmu-mosei-dataset/ (accessed on 8 June 2025). The CH-SIMS dataset, a Chinese MSA dataset with multi-label labels, is available for download through https://github.com/thuiar/MMSA (accessed on 8 June 2025).
